# Spotlight on the New Natural Surfactant Flooding in Carbonate Rock Samples in Low Salinity Condition

**DOI:** 10.1038/s41598-018-29321-w

**Published:** 2018-07-20

**Authors:** Mohammad Ali Ahmadi, Seyed Reza Shadizadeh

**Affiliations:** 1 Department of Chemical and Petroleum Engineering, University of Calgary, Calgary, Alberta Canada; 2 Department of Petroleum Engineering, Petroleum University of Technology, Abadan, Iran

## Abstract

Recently, utilization of surfactant for EOR purposes in carbonate petroleum reservoirs has received the attention of many researchers. Surfactants generally appear to improve oil production through wettability alteration and reduction of interfacial tension (IFT) between oil and water phases. Loss of surfactant due to adsorption process is considered as an unfavorable phenomenon in surfactant flooding while conducting an EOR operation. In this study, a new plant-derived surfactant, called Zyziphus Spina Christi (ZSC), with various magnitudes of salinity is employed. The adsorption behavior of this surfactant is investigated using the conductivity approach to explore the impacts of salt concentration on adsorption rate through batch tests. Core flooding tests are also conducted to study the effects of surfactant/salinity on recovery factor and relative permeability. Employing the kinetics and isotherm models, MgCl_2_ and KCl exhibit the greatest and lowest influence on the adsorption phenomenon, respectively. It is also concluded that the pseudo-second order kinetics and Freundlich isotherm model can satisfactorily describe the adsorption behavior of the surfactant onto carbonates in the presence of salt for the kinetics and equilibrium tests conditions, respectively. According to the production history, it is found that increasing surfactant concentration leads to a considerable increase in oil relative permeability and consequently improvement of oil recovery.

## Introduction

About 60% of whole oil reservoirs across the world are in the category of carbonate reserves^1^. However, the oil production contribution of such reservoirs is less than 50% (almost 40%) of worldwide oil production^2,3^. In other words, during primary oil production and water flooding processes, a significant part of the oil in place remains inside the pores of carbonate rocks due to heterogeneities as well as deprived sweep efficiency that leads to unswept and/or bypassed oil. Having low recovery factor for carbonate settings, various EOR EOR techniques such as low salinity water injection^4,5^, surfactant flooding^6^, polymer-surfactant (SP) injection^7^, alkaline-surfactant-polymer^8^ (ASP) injection in this type of petroleum formations have become interesting to research centers and oil companies in the recent years. Indeed, the main objectives of employing low salinity water and/or surfactant is to reduce the IFT parameter and also turn the wettability from oil-wet to neutral or water-wet^4–8^. The surfactant is generally mixed with water during the injection processes. Other chemicals such as water-soluble polymer and alkaline may also have contributions in oil production while operating water/surfactant injection. Adsorption fate of surfactant is the main concern in this type of production methods that limits the use/application of various surfactants for different oil reservoirs. A number of researchers have focused on this important issue^9,10^. For instance, Rodriguez^9^ studied the adsorption of surfactants on carbonate rock samples taken from an oil reservoir in Saudi Arabia^9^. The influences of surfactant charge and flow rate were discussed in this investigation where the surfactant concentration and temperature were decided to be 0.25 wt% and 107 °C, respectively^9^. According to the results, when the flow rate increases, the amount of adsorption will decrease. Besides the experimental works, several valuable analytical models have been developed to determine the adsorption behavior of surfactants from mathematical viewpoint^10–12^. Such models provide very deep understanding regarding adsorption phenomenon of surfactants onto the solid surfaces. Coupling these analytic models and sensitivity analysis method could help us to figure out how special surfactant behaves in a system containing surfactant, oil, and reservoir rock; using these approaches lead to better whole picture of wettability alteration using surfactant injection due to different mechanisms including wettability gradient, aqueous drop autophobing, and chemical reactions^10–12^.

For most of the flooding processes, the wettability parameter in carbonates plays an important role in oil production^11,12^. Since changing wetness from oil wet to water wet can enhance recovery factor while waterflooding, employing surfactant for this alteration appears to be interesting to researchers in this area^13–15^. For example, the wettability alteration of Yates rock through using ethoxy alcohol surfactants was studied by Vijapurapu and Rao^13^. Based on a research study carried out by Seethepali *et al*.^14^, it was concluded that the wetness of a calcite surface is changed to intermediate/water-wet wettability if an anionic surfactant in the presence of Na_2_CO_3_ is employed^14^. They also claimed that the tested anionic surfactants show better performance, in contrast with the cationic surfactants^14^. The influences of water/oil ratio, surfactant concentration, electrolyte concentration on wetness change were also investigated by Zhang *et al*.^15^. According to their results, alkaline/anionic surfactant systems are able to change the oil-wet calcite surface to an intermediate or water-wet medium. On the other hand, the existence of sodium carbonate can lower the amount of anionic surfactants adsorbed onto a dolomite surface, appreciably^15^.

In general, the key parameters influencing the phase behavior of oil/brine/surfactant and also adsorption process are the brine salinity and formation heterogeneity^6,16–18^. Adams and Schievelbein (1987)^6^ reported the information about a real case that deals with the surfactant flooding into a carbonate formation^6^. The case was a pilot-scale project that included two well pairs drilled in a San Andreas dolomite oil reservoir, located in West Texas. Using the tracer tests, it was found that the magnitudes of residual oil saturation for those two well pairs are about 7.5% and 18%, respectively^6^. According to their research, reservoir heterogeneity and salinity as two main characteristics have a considerable contribution to the performance of surfactant flooding^6^. Winsor^16^ also introduced three various kinds of systems, depending on the value of salt concentration; namely, (a) the surfactant exhibits a high solubility in the aqueous phase, but weak solubility in oily system when the salt concentration is low, (b) the surfactant is reasonably soluble into the oleic phase; however it shows poor solubility in the aqueous phase where the salinity is at an elevated level, (c) three phases consisting of excess brine, excess oil, and micro-emulsion are maintained when the brine concentration holds an intermediate value^16–18^. In the last category, micro-emulsion is a homogeneous phase including oil, brine, and surfactant at thermodynamic equilibrium with other phases^16–18^.

Ahmadi and Shadizadeh^19^, Ahmadi and Shadizadeh^20^, and Ahmadi and Shadizadeh^21–23^ studied the effect of surfactants on production history as well as the adsorption kinetics and isotherm behavior of two new surface active agent, entitled Zyziphus Spina Christi (ZSC), through static and dynamic tests onto the carbonate rock samples taken from Iranian oil fields when the salt concentration is kept constant^19–23^. Also, those studies systematically investigated important aspects such as the impact of nano-particles on adsorption behavior of ZSC onto carbonates while conducting a thermodynamic analysis of the adsorption process^22,23^.

Highlighting the contribution of this work, it exhibits important contribution compared to the studies focusing on the same topic, as a new natural surfactant is introduced and effect of salinity due to various types of salts is investigated on the adsorption behavior in terms of kinetics and equilibrium values, and adsorption mechanism. On the other hand, both static and dynamic tests are implemented to obtain the amount of adsorption and residual water saturation at isotherm conditions. To our knowledge, such a study is not available in the literature.

The current research study involves different salts such as KCl, NaCl, and MgCl_2_ in the test mixture in order to understand the effects of salt on the adsorption process. To determine important parameters such as CMC and adsorption density, the conductivity methodology was applied during the adsorption experiments. Depletion solution technique was also used to study the effects of adsorbate dose and salt on the adsorption density at the thermodynamic condition (P = 1 atm and T = 28 °C). To describe the adsorption behavior and mechanism, the most common adsorption isotherm and kinetics models were evaluated to know which models have a better agreement with the experimental data. In addition, core flooding experiments were performed in this study to exhibit the effectiveness of adding this natural surfactant to the brine while implementing waterflooding for EOR purpose.

## Theory: Adsorption Models

In this section, four common adsorption isotherm (equilibrium) models and three main adsorption kinetics models examined are briefly illustrated to understand adsorption behavior of ZSC surfactant at various salt concentrations.

### Langmuir Isotherm (Equilibrium)

Assuming that the adsorption surface is totally homogenous, Langmuir introduced the following relationship^19–24^:

1 $${q}_{e}=\frac{{q}_{o}\,{K}_{ad}\,{C}_{e}}{1+{K}_{ad}\,{C}_{e}}$$

where *q*_*e*_ represents the equilibrium adsorption density (mg/g), *C*_*e*_ stands for the equilibrium surfactant concentration, *q*_*o*_ is the maximum magnitude of adsorption, and *K*_*ad*_ introduces the Langmuir adsorption constant.

Taking a look at Equation (1), a plot of 1/*q*_*e*_ against 1/*C*_*e*_ results in a straight line with a slope of (1/*C*_*e*_ × *K*_*ad*_) and a constant intercept of (1/*C*_*e*_).

### Freundlich Isotherm (Equilibrium)

This model was developed to demonstrate the adsorption phenomenon if it is assumed that the adsorbent has a heterogeneous surface being made of various types of adsorption locations^25^. The Freundlich isotherm is described by the following equation^19–23,26^:

2 $${q}_{e}={K}_{f}\,{C}_{e}^{1/n}$$

in which, 1*/n* and *K*_*f*_ stand for the Freundlich constants. The Freundlich constant (1*/n*) refers to the adsorption intensity of the adsorbent, as well. When 0.1 < 1/*n* < 0.5, adsorption would be favorable; 0.5 < 1/*n* ≤ 1, adsorption process would be easily attainable; *1*/*n* > 1, adsorption would be almost impossible^26^.

### Temkin Isotherm (Equilibrium)

The linear form of the Temkin isotherm is presented as below^19–23,25,27^:

3 $${q}_{e}=B\,\mathrm{ln}\,{K}_{t}+B\,\mathrm{ln}\,{C}_{e}$$

where *K*_*t*_ represents the equilibrium binding constant related to the maximum binding energy (L/mg), and *B* is called Temkin constant which corresponds to the heat of adsorption^25,27^.

### Linear Isotherm (Equilibrium)

Based on the assumption of Henry equation, a linear relationship is defined between surfactant concentration and adsorption density as follows^19–23,25,27^:

4 $${q}_{e}={K}_{H}\,{C}_{e}$$

in which, *K*_*H*_ is a constant with the unit of L/m^2^.

### Pseudo-First-Order Model (Kinetics)

The general form of this kinetics model for adsorption is typically written as follows^25,27–29^:

5 $$\mathrm{log}\,({q}_{e}-{q}_{t})=\,\mathrm{log}\,({q}_{e})-\frac{{K}_{1}}{2.303}t$$

in which, *q*_*t*_ refers to the amount of adsorbed surfactant (mg/g of rock) at a certain time(*t*), and *K*_*1*_ is the rate constant of the pseudo-first-order model^25,27–29^.

### Pseudo-Second Order Model (Kinetics)

The simplified form of the pseudo-second-order model is expressed as the following^25,27–29^:

6 $$\frac{t}{{q}_{t}}=\frac{1}{{q}_{e}\,{K}_{2}}+\frac{t}{{q}_{e}}$$

where *K*_2_ introduces the rate constant of the pseudo-second-order model.

### Intra-Particle Diffusion Model (Kinetics)

The amount of surfactant adsorption is a linear function of square root of time in this adsorption kinetics model on the basis of the study conducted by Weber and Morris (1963), as given below^27–29^:

7 $${q}_{t}={K}_{i}\,{t}^{0.5}$$

In Equation (7), *K*_*i*_ is the symbol for the rate constant of the intra-particle diffusion model.

Technical readers are encouraged to study these documents for further information on the adsorption isotherms and kinetics^19–29^.

## Materials

### Surfactant

Zizyphus Spina Christi (ZSC) is a well-liked tree which is available in the Middle East countries such as Jordan, Iran, Iraq, and Egypt^29–31^. Saponin which is a natural and bio-surfactant is extracted from the leaves of ZSC^29–31^. ZSC leaves were gathered from Khuzestan province which is located in the south of Iran. Then, the saponin was extracted and dried using the spray dryer technique. It should be noted that the extracted powder from the leaves contains Saponin and Flavonoids with light brown color which is soluble in water and alcohol. Saponin is a biodegradable natural surfactant. Properties of the ZSC surfactant are given in Table 1^19–23,29–31^.

**Table 1:** Properties of the natural surfactant (ZSC)^19–23^.

Name of Substance	Zyziphus Spina Christi
Used Piece	Leaves
Preparation	Spray Drier
Description	Fine Powder
Color	Brown
Magnitude of pH (10% Solution)	5.9–6.0
Applications	Medicine, Enhanced Oil Recovery

### Adsorbent

The carbonate rock samples used in the current study have been supplied from one of the northern Persian Gulf oil fields in the south of Iran. Samples were broken to small pieces by a jaw crusher and then grounded. After that, the samples obtained were dried with air for about 24 hours by an oven at the temperature of 105 °C. As the particle size of adsorbent (e.g., rock) had a broad distribution, the dried carbonate rocks were filtered by the sieves of No. 50 and No. 70 to achieve the particles in the range of 210–297 microns^19–23^.

### Oil sample

The crude oil employed for the dynamic tests is taken from Naft-Shahr oil field (a shared oil field between Iran and Iraq), situated at the northern of Persian Gulf. The oil-bearing formation includes fractured carbonate Asmari reservoir and Anhydrite Kalhor reservoir. Table 2 lists the main characteristics of the crude oil.

**Table 2:** Properties of the oil employed in this study^23^.

Component	Oil Composition	
	mol %	wt %
C_1_	0	0
C_2_	0.43	0.069465
C_3_	0.89	0.21085
IC_4_	0.9	0.28104
NC_4_	1.34	0.41843
IC_5_	1.21	0.46902
NC_5_	4.77	1.849
C_6+_	90.45	96.7
H_2_S	0.01	0.001831
N_2_	0	0
CO_2_	0	0
Mw of C_6+_ (g/gmol)	199	
SG of C_6+_	0.8181	
Oil Density	43°API	

*Mw* and *SG* stand for the molecular weight and specific gravity, respectively.

## Experimental Methodology

This section explains the main stages of static adsorption experiments (e.g., solution preparation and CMC measurement) and dynamic core flooding tests (e.g., measurement of relative permeability) through a brief, but clear manner. In general, the experiments were performed at least twice. Therefore, we had 1–2 replicates for each run. Based on the statistical analysis, the relative error percentage was in the range of 1.0–3.0% for all test trials as the conductivity measurements exhibit fairly low errors. The standard deviation was also between 0.5–1.3% of the replicates mean for the tests. Therefore, the average values of the replicates for each particular condition are used in this study for the purpose of calculation, comparison, and discussion.

### CMC Measurement (Static Tests)

To determine the critical micelle concentration (CMC) of the ZSC solution, the conductivity approach was used^7–9,19–23^. Based on the CMC value, the surfactant concentration varied between 1000 and 80000 ppm throughout the tests. More information on CMC determination is found in these documents (e.g., Ahmadi and Shadizadeh, 2012 & 2013)^19–23^.

### The amount of Surfactant Adsorption (Static Tests)

Known weights of the crushed carbonate rocks were added to a ZSC solution with certain concentrations of salt and ZSC in the bottles. The bottles were then shaken under a controlled condition (e.g., constant T and P). The conductivity tests were carried out to determine the surfactant concentration in the solutions before and after adsorption process. Given the concentrations, the adsorption density is obtained as the following^19–23^.

8 $$q=\frac{({C}_{ini}-{C}_{fin})\,{V}_{sol}}{1000\,{m}_{car}}$$

in which,

*q* is the surfactant adsorption on rock surface (mg/g-rock);

*V*_*sol*_ is the total volume of solution in original bulk solution (mL);

*C*_*ini*_ is the surfactant concentration in initial solution before equilibrated with the carbonate rock (ppm);

*C*_*fin*_ is the surfactant concentration in aqueous solution after equilibrated with the carbonate rock (ppm); and

m_car_ is the total mass of the crushed carbonates (g).

### Determination of Relative Permeability (Dynamic Tests)

At the first stage, the porous core placed in a core holder was saturated with water, and then the reservoir crude oil was injected as the displacing fluid at a constant flow rate of 0.1 cm^3^/min. The operating conditions were a pressure of 1500 psi and a temperature of 100 °C. A graduate cylinder was also used to accumulate (and measure) the produced fluids while operating the tests. The required parameters such as pressure difference between the inlet and outlet, and volumes of oil and water phases were being measured at certain time periods as long as the oil was produced. Considering three main steps; namely, [a) only oil exists in the output stream, b) the first droplet of water is observed in the outlet, and c) no more oil is produced from the core] Equations (9) and (10) were employed to calculate water saturation at each time interval and also the residual oil saturation, respectively, as follows:

9 $${S}_{w}=\frac{({V}_{wi}+{V}_{op})}{PV}$$

10 $${S}_{or}=\frac{({V}_{oi}-{V}_{op})}{PV}$$

in which, *S*_*w*_ represents the water saturation, *S*_*oi*_ denotes the residual oil saturation, *PV* stands for the pore volume of the core sample (cm^3^), and *V*_*wi*_ is the initial volume of water in the core sample (cm^3^). In addition, *V*_*oi*_ and *V*_*op*_ are the initial volume of oil and the volume of oil produced after flooding in the core sample (cm^3^), respectively.

Given the flow rates of oil and water, their viscosities, core length, and pressure difference, the values of relative permeability for oil and water are obtained.

## Results and Discussion

The petroleum reservoirs commonly contain a variety of salts (e.g., NaCl, KCl and MgCl_2_), because (1) gas and oil are frequently associated with salt deposits and (2) the connate water (or/and initial water) includes different kinds (and content) of salts. Depending on the rock types and reservoir fluids, low and high concentrations of salt may exist in the petroleum formations. On the other hand, the salinity of water used for injection into oil reservoirs is generally altered from moderate to high levels.

Adsorption tests of aqueous solutions of ZSC were implemented in this study. This environmental friendly-surfactant was used at different conditions that involve various salt concentrations. This section discusses the effects of the most important factor contributing to the adsorption process as well as variation of relative permeability of oil and water phases at different surfactant concentrations.

### Rocks Composition

**Figure 1:**
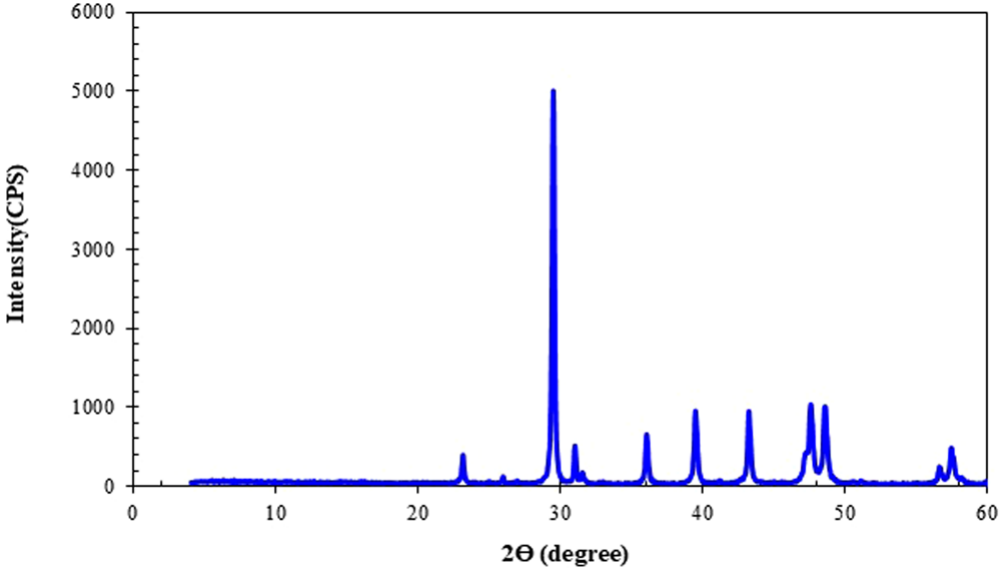
XRD of the crushed carbonate rock [Note: CPS denotes “Counts Per Second”].

**Figure 2:**
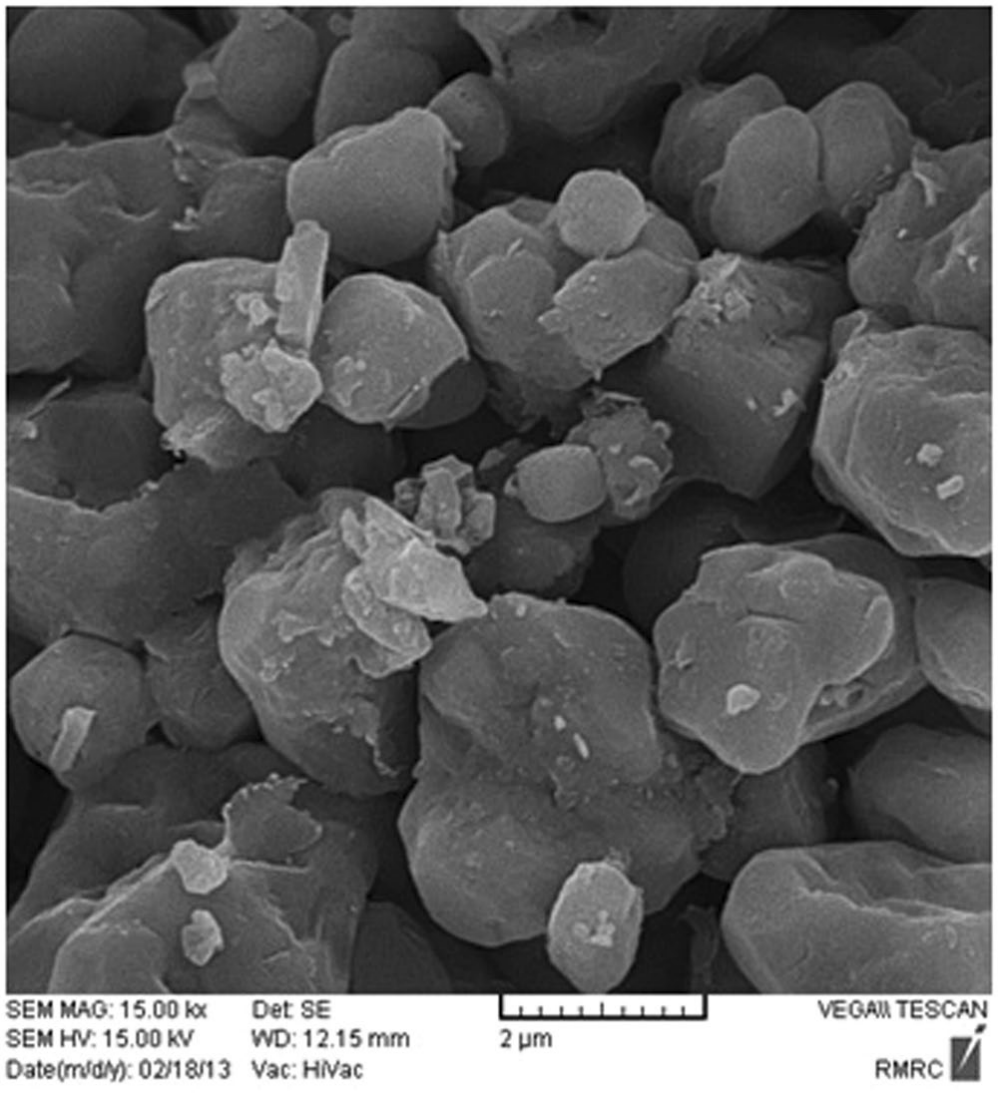
SEM of the carbonate rock.

X-ray diffraction (XRD) was utilized to determine the mineral composition of the carbonate samples. The results obtained from this technique are shown in Fig. 1. In addition, Scanning Electron Microscopy (SEM) provides the Supplementary Information which includes topography as well as the composition of the rock. Thus, the SEM technique can be used to qualitatively confirm the outcomes of XRD method. The SEM data are demonstrated in Fig. 2. Based on these two different methodologies, it was concluded that dolomite is the main component in the carbonate samples.

### CMC Parameter

**Figure 3:**
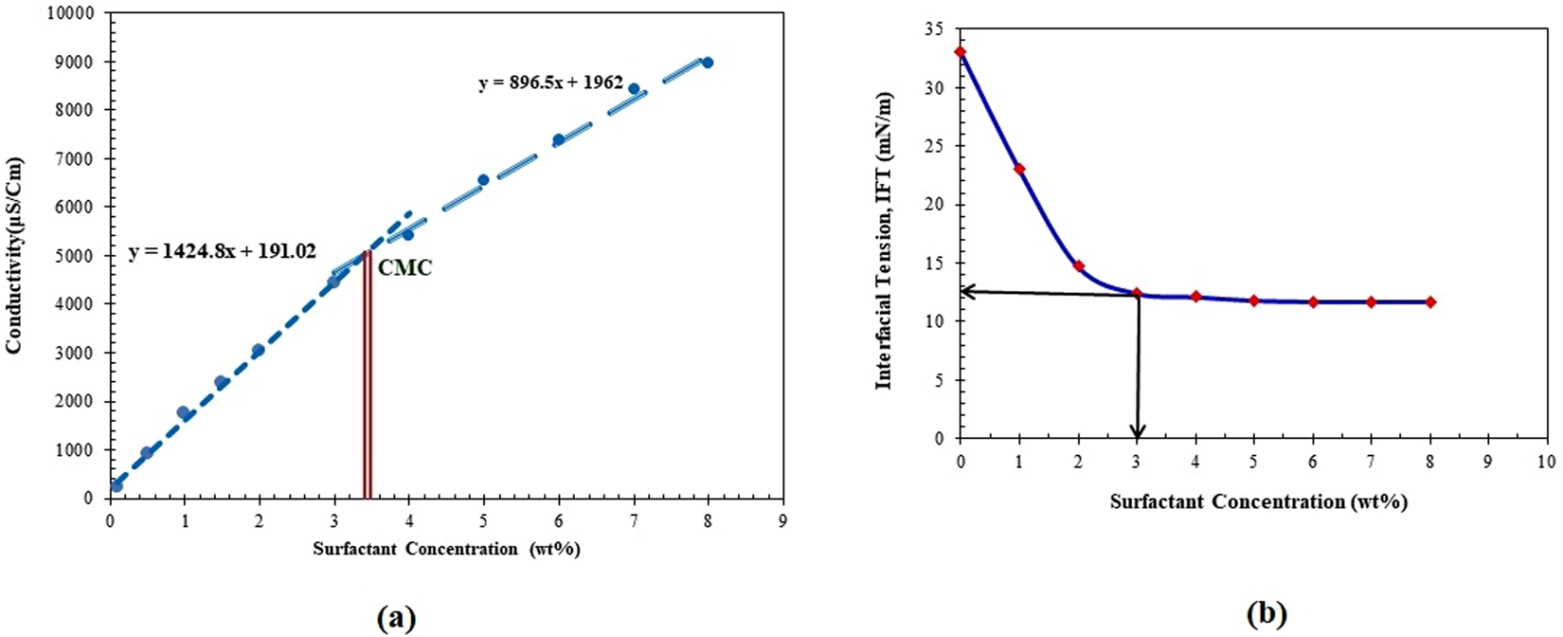
(**a**) Conductivity of the surfactant versus surfactant concentration. (**b**) Interfacial Tension (IFT) as a function of ZSC concentration^19–23^.

The results of the conductivity method are depicted in Fig. 3a^19–23^. The method is clearly demonstrated to obtain the CMC parameter for the case under study. Another technique to determine is plotting surface tension versus surfactant concentration. The first point in Fig. 3b that exhibits the minimum surface tension is attributed to CMC parameter after which the magnitude of surface tension remains almost unchanged. The CMC is determined to be at a surfactant concentration of about 3.4 and 3.0 wt%, according to Panels a and b of Fig. 3, respectively. As clear from Fig. 3, a relatively good agreement is noticed between these two deterministic techniques.

### Adsorption Kinetics

**Figure 4:**
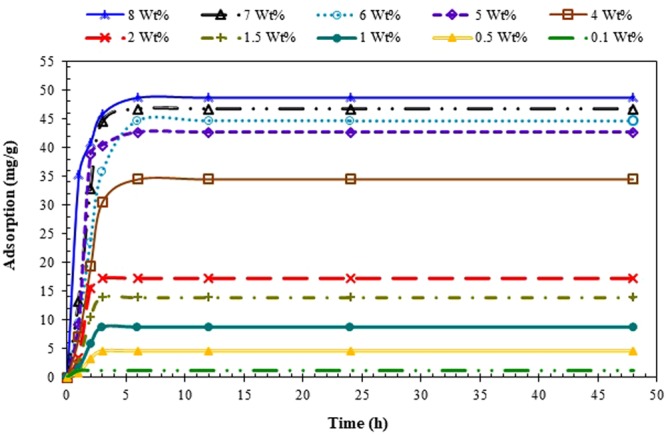
Dependency of surfactant concentration on adsorption and equilibrium time^19,22^.

**Figure 5:**
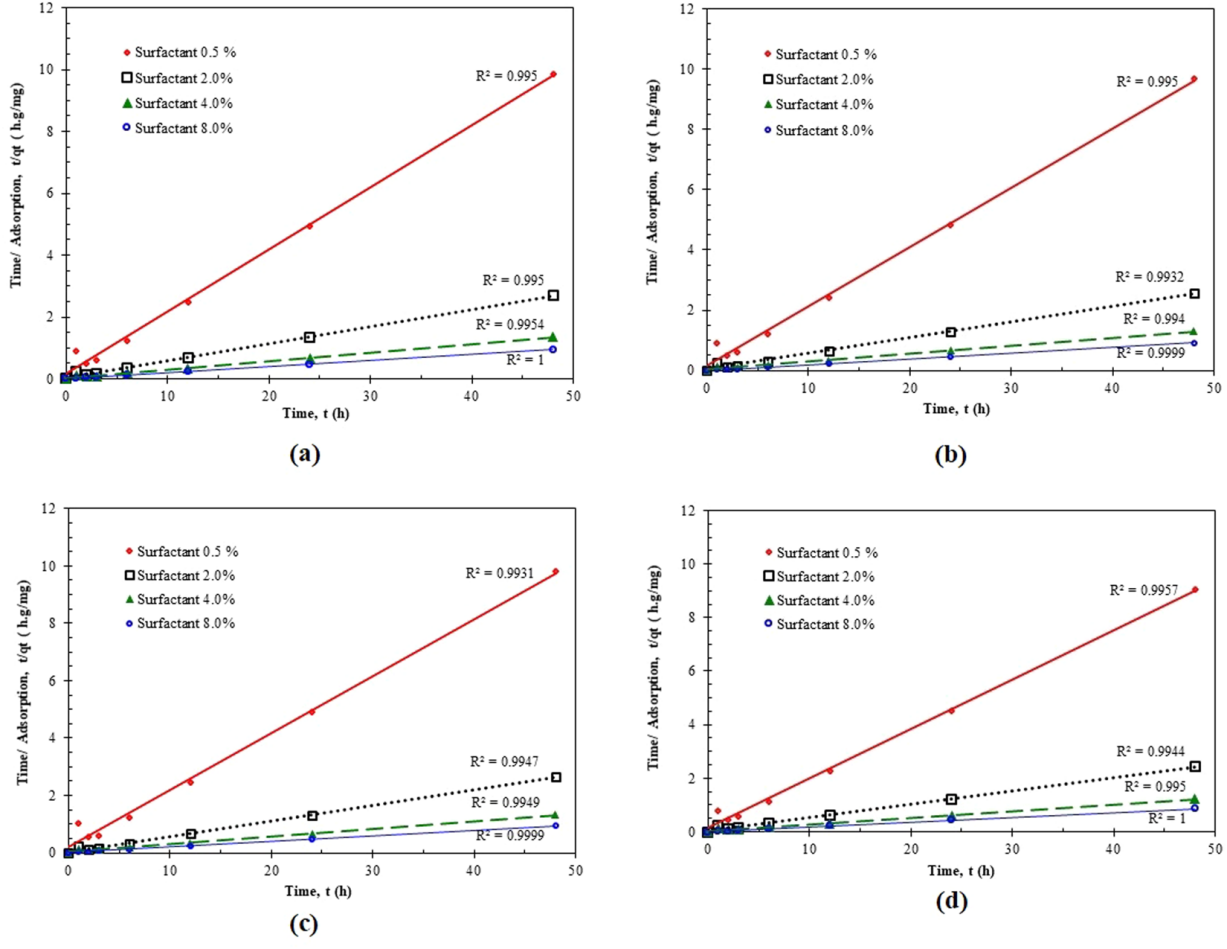
Experimental data fitted to the pseudo-second order kinetic model for ZSC adsorption by carbonate samples at various salt and surfactant concentrations.

The adsorption kinetics tests (e.g., batch) of a particular surfactant appear to be important for the design of a surfactant with an appropriate concentration for practical surfactant EOR processes. To find out the kinetic behavior of the adsorption process, the adsorption experiments were performed seven times including 1, 2, 3, 6, 12, 24 and 48 hours. Using the experimental data collected for the case under study, the kinetic adsorption trend clearly conveys the message that the surfactant solution in contact with crushed carbonate rock samples reaches to the equilibrium condition after 6 hours, as shown in Fig. 4. The applicability of all three common kinetics models (e.g., pseudo-first order model, pseudo-second-order model, and intra-particle diffusion model) described in the Theory Section was examined in the current study. The general procedure is that the real data are fitted with the models and then the model that exhibits the highest correlation coefficient (*R*^2^) and minimum error percentage is selected as the best kinetics equation. Due to the space limitation, the results for inappropriate models are not presented in the paper and only the proper model is described here. As shown in Fig. 5, high values of (*R*^2^) attained for the fitted pseudo-second order kinetics imply that adsorption of ZSC surfactant onto carbonates obeys the pseudo-second-order model.

### Adsorption Isotherms

**Figure 6:**
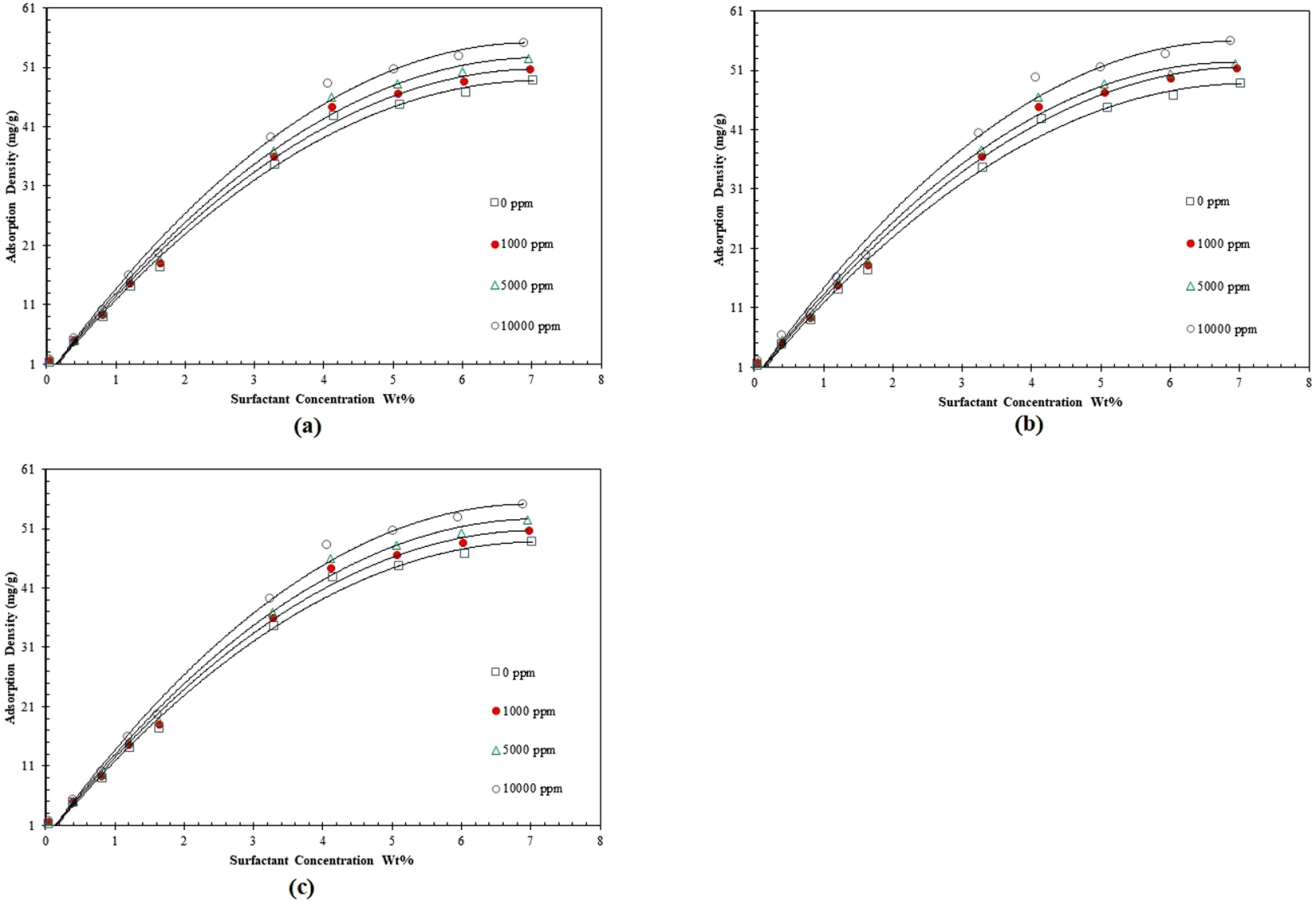
Adsorption density versus equilibrium surfactant concentration at different concentrations of, (**a**) KCl, (**b**) NaCl, (**c**) MgCl_2._

Knowledge about adsorption process in porous media plays a crucial role in design and optimization of an adsorption system. Adsorption density of the natural surfactant against the equilibrium surfactant concentration at different salinities is demonstrated in Fig. 6. As clear from Fig. 6, adding salt causes an increase in the adsorption density. Further elaboration is found in the section related to the effect of salt concentration.

In this paper, Langmuir, Freundlich, Temkin, and linear isotherm models are listed to address important aspects of the surfactant loss such as adsorption density. The document “Supporting Information” presents the adsorption of ZSC onto carbonate rocks based on various adsorption isotherms (those ones which were confirmed not to be appropriate to predict the amount of ZSC adsorbed on the carbonates).

**Figure 7:**
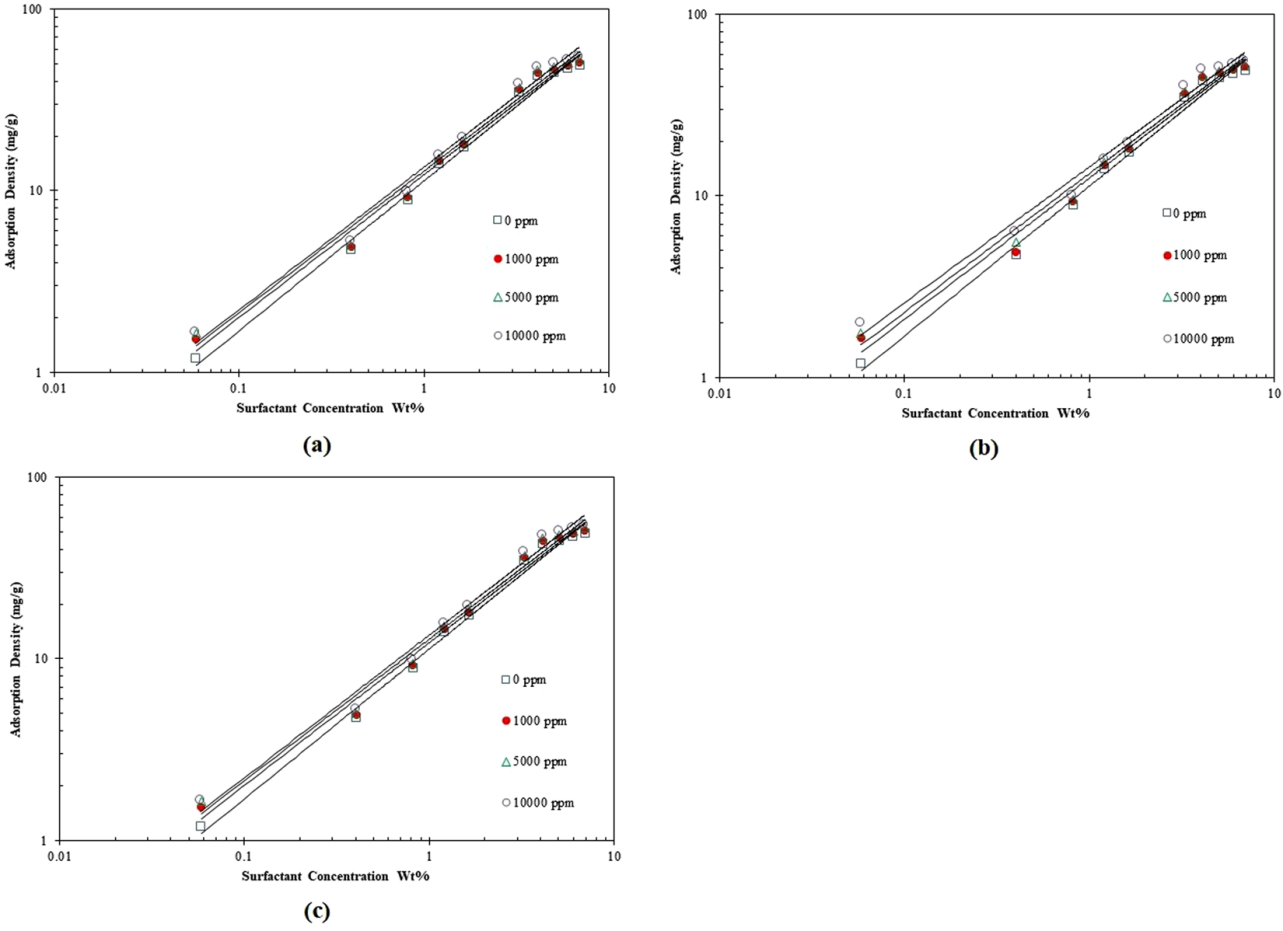
Freundlich isotherm model of natural surfactant adsorption onto carbonates at different concentrations of, (**a**) KCl, (**b**) NaCl, (**c**) MgCl_2_.

The Freundlich model being valid for a series of data represents the heterogeneity of the sorbent surface. The magnitude of the Freundlich exponent (1/*n*) stands for the capacity of the adsorbent/adsorbate system^25–27^. Based on the data shown in Fig. 7, the Freundlich isotherm parameters are computed as listed in Table 3. As the values of correlation coefficient (*R*^2^) indicate, the Freundlich isotherm is able to forecast the surfactant loss with reasonable accuracy. It conveys the important message that the adsorption of the ZSC onto carbonate rock samples is not homogenous and mono-layered.

**Table 3:** Parameters of the Freundlich adsorption model used in this study.

MgCl_2_ (ppm)	Correlation	*R* ^2^	*K*_*F*_	*1/n*
0	q_e_ = 11.304 (Ce)^0.8231^	0.9926	11.304	0.8231
1000	q_e_ = 12.777 (Ce)^0.7874^	0.9875	12.777	0.7874
5000	q_e_ = 14.08 (Ce)^0.7378^	0.9857	14.08	0.7378
10000	q_e_ = 15.215 (Ce)^0.7434^	0.9881	15.215	0.7434
**KCl (ppm)**				
1000	q_e_ = 12.278 (Ce)^0.7861^	0.9881	12.278	0.7861
5000	q_e_ = 12.855 (Ce)^0.78^	0.9869	12.855	0.78
10000	q_e_ = 13.517 (Ce)^0.7857^	0.9879	13.517	0.7857
**NaCl (ppm)**				
1000	q_e_ = 12.569 (Ce)^0.7785^	0.9862	12.569	0.7785
5000	q_e_ = 13.284 (Ce)^0.7633^	0.9876	13.284	0.7633
10000	q_e_ = 14.47 (Ce)^0.7487^	0.9863	14.47	0.7487

**Figure 8:**
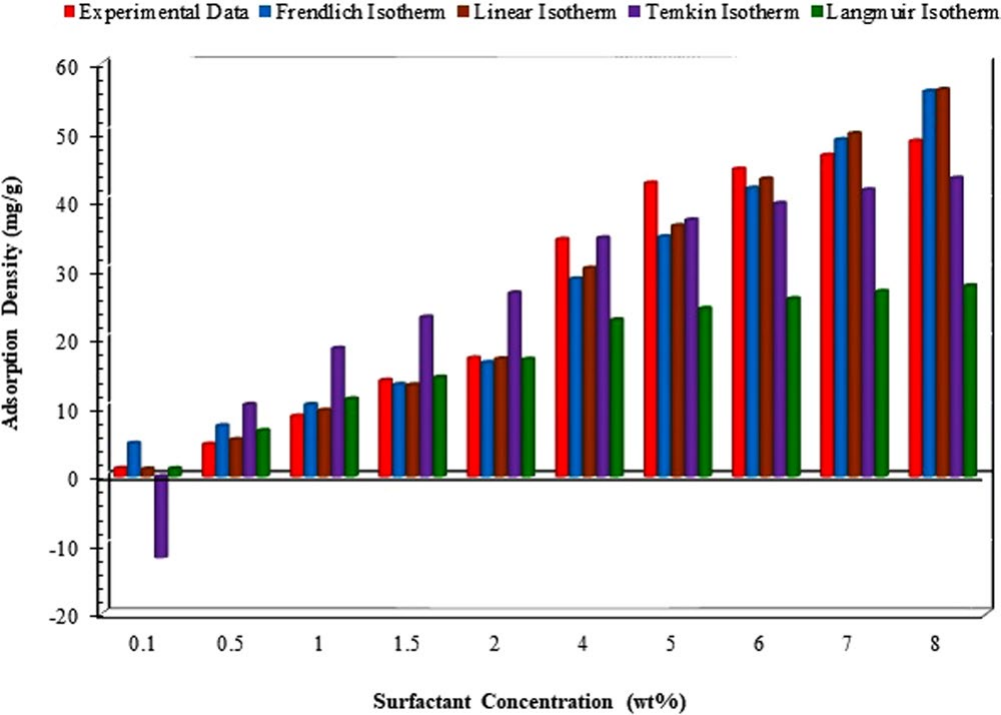
Comparison of the models outcomes and corresponding adsorption data versus surfactant concentration.

According to the values of *R*^2^, the experimental data obey the Freundlich and Langmuir isotherms (See Supporting Information) better than the other isotherms. For the comparison purpose, Fig. 8 includes the results for adsorption density of ZSC based on all isotherm models in aqueous solution without salt content. According to this Figure, Freundlich and Linear isotherms are appropriate to predict the surfactant loss due to adsorption, though the Freundlich model shows a higher performance as a predictive tool for the adsorption of ZSC onto carbonate samples. However, as it is clear from Fig. 8, the Temkin isotherm fails to appropriately describe the adsorption mechanism of the natural surfactant at some surfactant concentrations. For example, it gives the negative value for the adsorption density (when the surfactant concentration is set to 0.1 wt %) which does not make a sense. Summarizing the results, the experiments show that the linear, Langmuir and Temkin equilibrium adsorption models are not suitable to explain the ZSC adsorption onto the carbonate rock faces, while the Freundlich equilibrium adsorption demonstrates an acceptable agreement between the real data and predicted outcomes so that *R*^2^ = 0.987 for CaCl_2_, *R*^2^ = 0.988 for KCl and *R*^2^ = 0.987 for NaCl were attained.

In summary, it should be noted that the most suitable isotherm for ZSC adsorption is selected based on the values of *R*^2^ and error percentage when all adsorption models are tested. The best fit of experimental data on the basis of the Freundlich model under equilibrium conditions also verifies the heterogeneous coverage of ZSC onto carbonate surface.

Generally speaking, as the adsorbent surface is heterogeneous and the adsorption in our case occurs due to ion exchange, the isotherm Freundlich model can simulate the adsorption process at equilibrium condition well. Supporting this statement, this isotherm model is favorable for ZSC adsorption, as high determination coefficient is attained (e.g., 0.99).

It is worth noting that the crude oil components can alter the amount or rate of adsorption, depending on the interaction between oil, water and surfactant, type of ions, and interfacial tension of oil/water system. As the current research work is focusing on important aspects of this matter in terms of adsorption kinetics and equilibrium for various oils with different characteristics (e.g., composition, physical properties, and so on). The experimental and modeling results for different wettability and oil samples will be published in the near future.

Temperature plays an important role in the adsorption of surfactant on carbonates, showing two main influences on the adsorption process. Increasing temperature will enhance the rate of diffusion of surfactant molecule across the external boundary layer and in the interior pores of the carbonate particles since the molecules movement increases and also the solution viscosity declines as the temperature increases. Furthermore, the temperature affects the equilibrium capacity of the carbonates, depending on whether the adsorption process is exothermic or endothermic. Since the adsorption of ZSC on the carbonates is an exothermic process, the adsorption capacity of this surfactant decreases with increasing temperature.

When physisorption of gasses or liquids over solids occurs, the amount of adsorption will increase if the pressure experiences an increase. This is because the adsorbate volume lowers during the adsorption process. It should be noted here that the effect of pressure is much stronger in gas adsorption cases, compared to liquid adsorption systems. The current study was conducted at the atmospheric conditions. Implementation of high pressure and temperature experimental runs would be a part of our future research work.

### Salt Concentration

**Figure 9:**
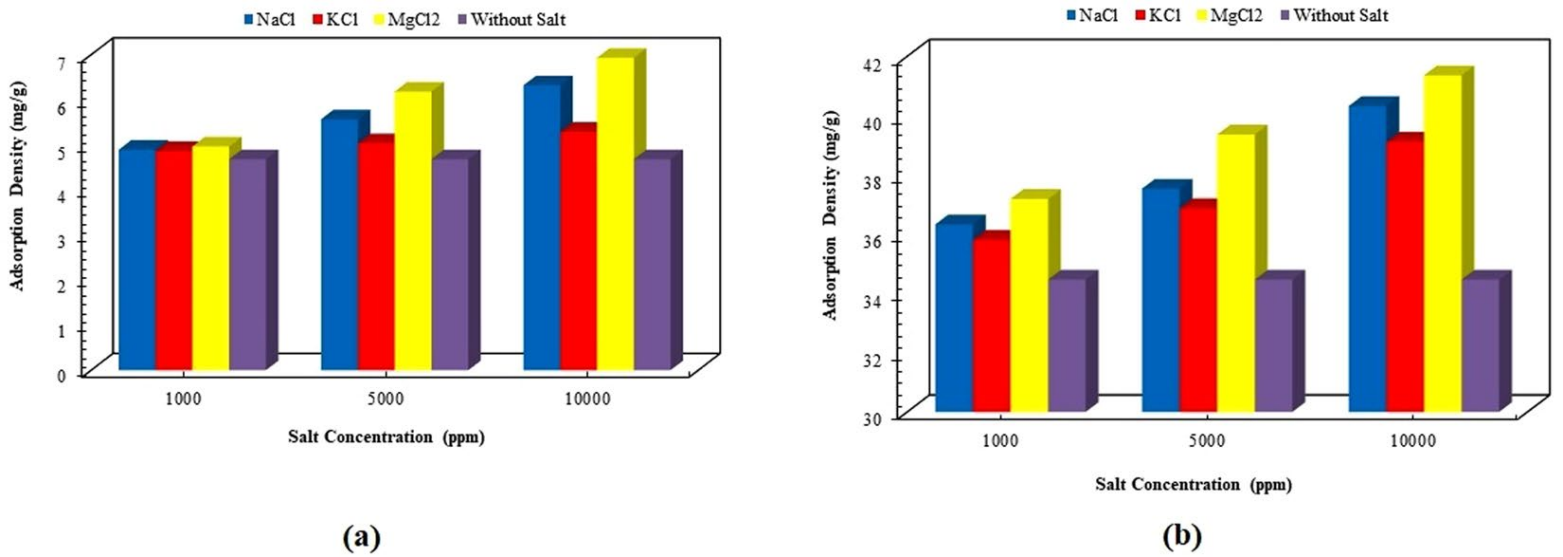
Effect of salt concentration on adsorption density of solution with the concentration of, (**a**) 0.5 wt%, (**b**) 4 wt%.

**Figure 10:**
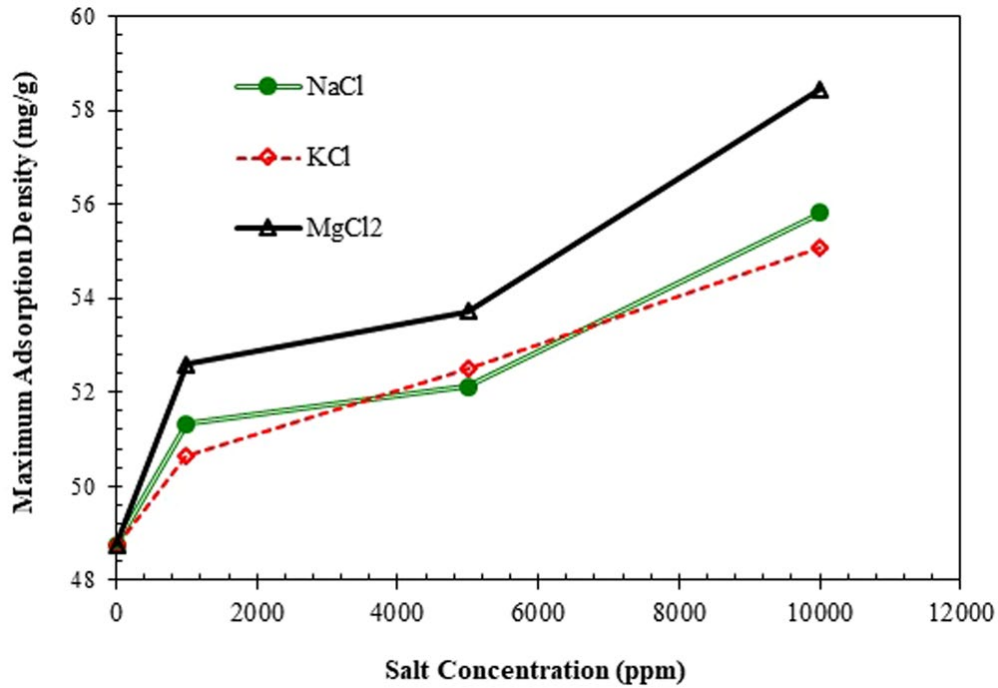
Effect of salt concentration on maximum adsorption density of the natural surfactant.

Figures 9 and 10 describe the influence of salt concentration on the amount of adsorption density for various surfactant concentrations. As clear from Fig. 9a, lower salt concentrations do not have a considerable effect on the adsorption behavior of ZSC. However, increasing salt concentration affects significantly the magnitude of adsorption density of the surfactant, particularly when MgCl_2_ and NaCl are present in the solution. Figure 9b also shows that the lowest influence of salts on the adsorption process is experienced for KCl, while MgCl_2_ undergoes the greatest impact on the adsorption. The same dependency in adsorption density with respect to salt concentration is observed at the solution concentration of 4 wt% (see Fig. 9, Panels a & b). Figure 10 demonstrates the effect of the mono-valent and divalent salts onto the maximum adsorption density of the surfactant when the surfactant concentration is 8 wt%. As illustrated in Fig. 10, at low and high ranges of salinity, the lowest and highest salt impacts are seen for NaCl and MgCl_2_, respectively. However; at intermediate values of salt concentration, KCl has the lowest effect on the maximum adsorption density for ZSC surfactant.

### Adsorption Mechanism

**Figure 11:**
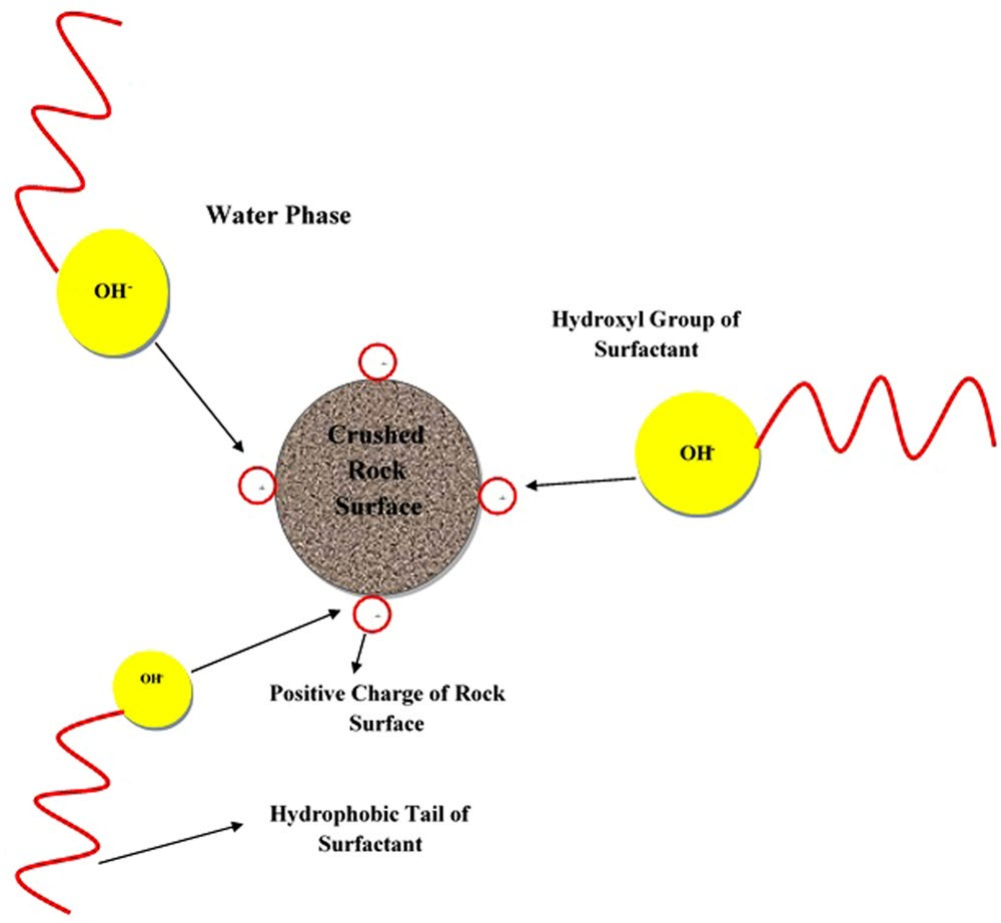
Schematic of adsorption mechanism of the natural surfactant onto carbonate rock surface.

**Figure 12:**
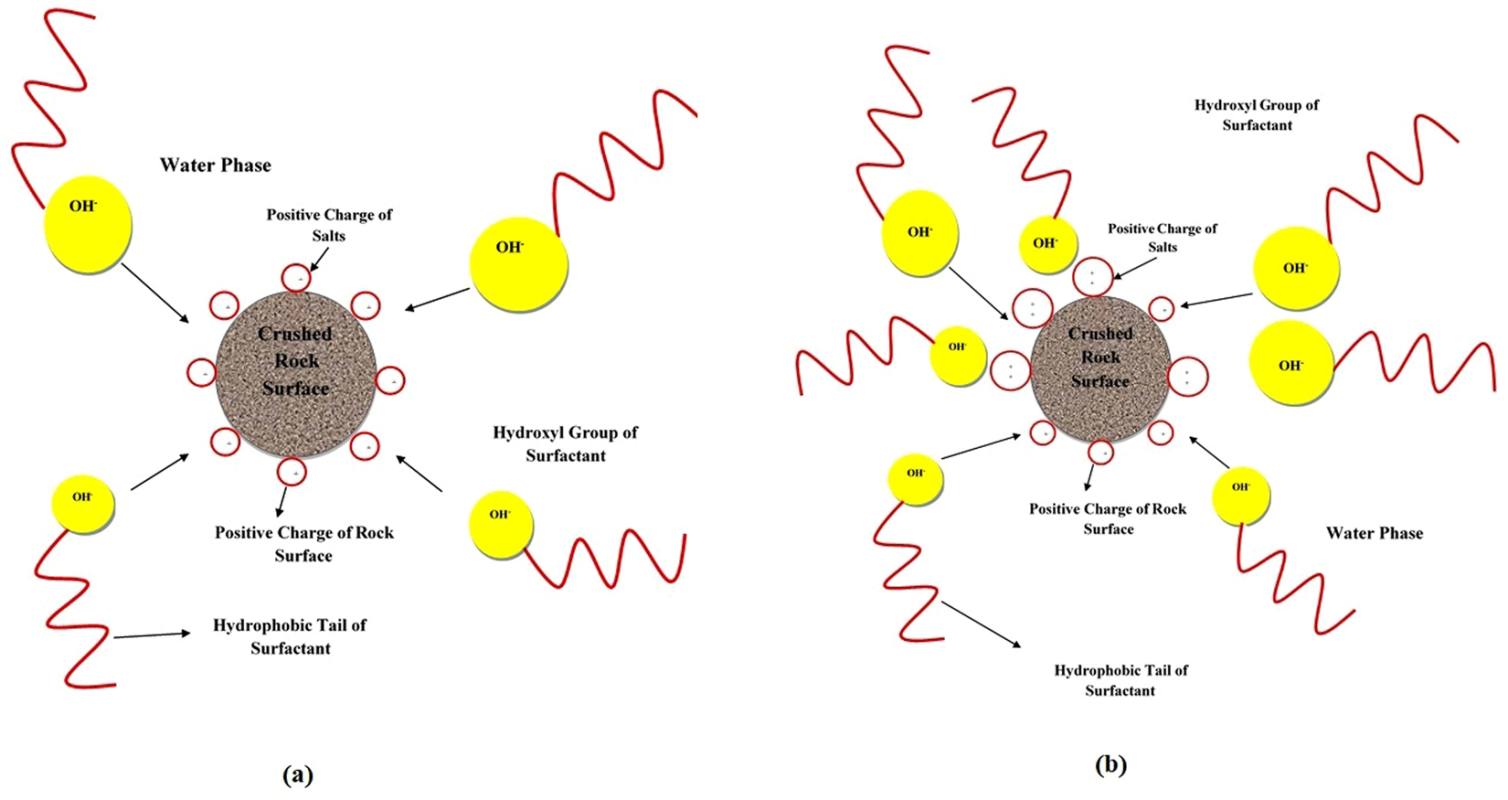
Schematic of adsorption mechanism of the natural surfactant onto carbonate rock surface in the presence of, (**a**) NaCl and/or KCl, (**b**) MgCl_2_.

To avoid excessive overlap between the current study and the previous works published by the authors, the general structure of the saponin is depicted in Appendix A. Based on Figure A1, saponin contains several hydroxyl groups, considering that the hydroxyl group has a negative charge^31,32^. On the other hand, the surface of carbonate that is mostly composed of dolomite contains positive charges. Hence, the most important mechanism for the surfactant adsorption is the electro-static attraction forces between negative charges of the hydroxyl groups and positive charges of the carbonate surfaces^25,27^. For further clarification, Fig. 11 demonstrates the adsorption process of the ZSC onto carbonate rock samples. Adsorption mechanism of the natural surfactant in the presence of mono-valent and divalent salts, respectively, is also described in Fig. 12. As shown in Fig. 12, a number of surfactant molecules adsorbed is increased by increasing the system charge. An increase in the salt concentration results in intensifying the charges of the carbonate rock surface, leading to loss of the surfactant due to the adsorption^7,25,27^. It should be noted here that the magnitude of charge plays a vital role in the amount of surfactant adsorbed onto rock faces during the adsorption process. This increase in the amount of charge for MgCl_2_ is higher than other mono-valent salts such as NaCl and KCl since MgCl_2_ releases Mg^2+^ ions in the system which has greater charges. In general, it is concluded that mechanism of adsorption process for ZSC is the electrostatic attraction force between the negative charge of hydroxyl groups of the surfactant and the positive charge of the carbonate rock surface. Therefore, adding salts causes an increase in the positive charge of the carbonate rock samples, leading to an increase in the attraction forces and the amount of surfactant loss.

Briefly describing the adsorption mechanism in a clear way, the water phase (brine) first sits on the rock surface which is hydrophilic. Therefore, the water is the interface between the rock solid and the surfactant. It implies that if the salt existing in the water phase contains more positive ions (e.g., Mg 2+), greater adsorption occurs between the positive ions and the hydroxyl group of the surfactant, resulting in the higher amount of the surfactant adsorbed in the process.

Discussing other important mechanisms that might happen in surfactant injection tests, salting out can play important role in adsorption processes for some particular surfactants. It is known that if salting out effect is dominant throughout the adsorption process, a portion of the surfactant should be precipitated^9,14^; however, no surfactant precipitation was noticed in the experiments. Hence, it can be concluded that the electrolytes improve the surface charge of the rock and consequently increase the amount of surfactant adsorbed onto the rock surfaces.

As a practical recommendation, it seems helpful to somehow treat water for salt removal before water injection is implemented. Regardless of the treatment cost, this strategy appears to be effective to reduce the surfactant adsorption, leading to improvement of oil production. Furthermore, if a specific additive is added to act as a barrier between the hydroxyl group of surfactant and the surface or/and groups with positive charges, it prevents attraction between the surfactant and the carbonate surface, resulting in a reduction of adsorption rate.

### Core flooding Tests

**Figure 13:**
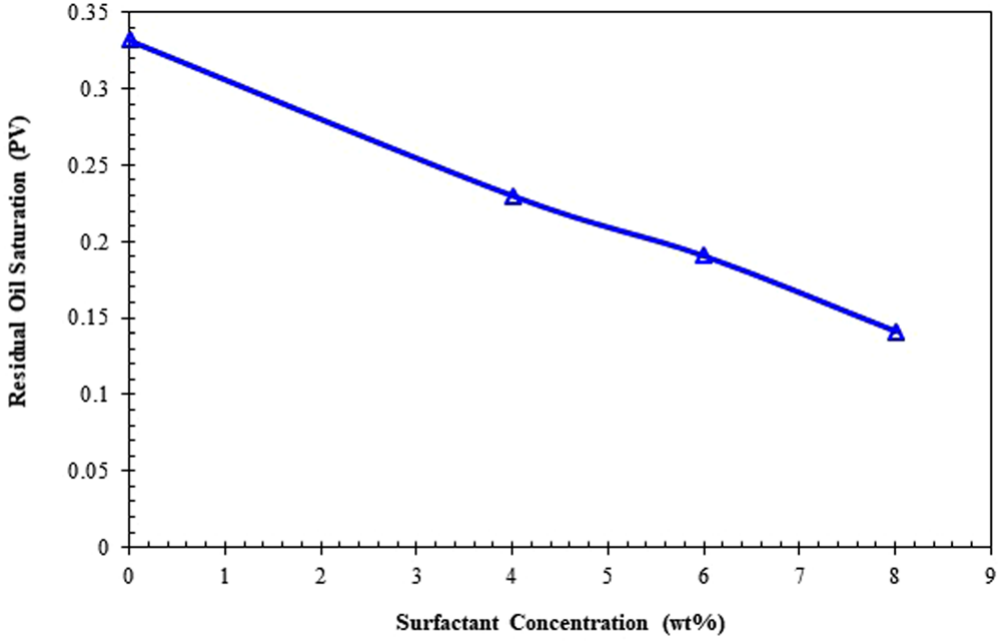
Residual oil saturation versus surfactant concentration.

**Figure 14:**
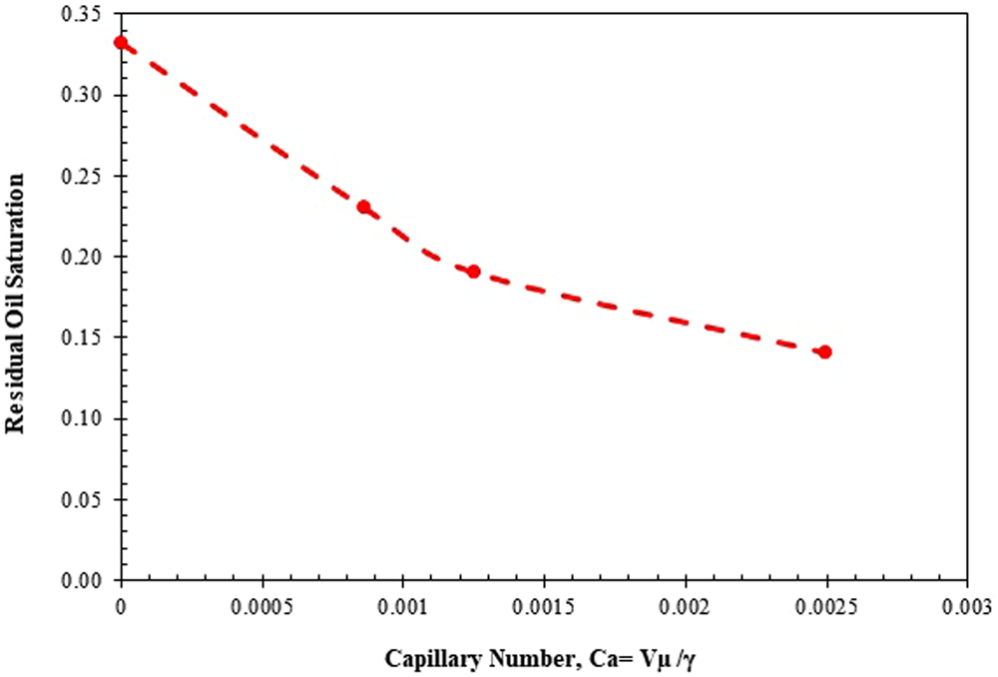
Variation of residual oil saturation with respect to capillary number [Ca stands for the capillary number, V is the Darcy velocity of displacing fluid, µ is the viscosity of displacing fluid, and γ is the interfacial tension].

Effects of the surfactant concentration and injection rate on residual oil saturation are demonstrated in Figs 13 and 14, respectively, on the basis of the data obtained from the displacement experiments. As clear from Fig. 13, the residual oil saturation is significantly decreased when the surfactant concentration in the aqueous phase increases. According to Fig. 14, increasing the capillary number (Ca = Vµ/γ), by an increase in injection rate (V) or/and reduction in the interfacial tension (γ) leads to decreasing the amount of residual oil saturation. For example, when the capillary number increases from 1.0 × 10^−4^ to 12 × 10^−4^ (while using 8 wt% surfactant), the residual oil saturation varies from 0.331898 to 0.140954. It is worth noting that the surfactant concentration will change along with the core length. Also, it is common to consider average residual oil saturation within the core while studying the effect of surfactant on oil recovery performance. Therefore, to have a proper value for the capillary number for each particular experiment, the average value of interfacial tension of oil/water system at input and output points is considered, though just 10–15% difference between input and exit interfacial tensions in terms of numerical value was noticed.

**Figure 15:**
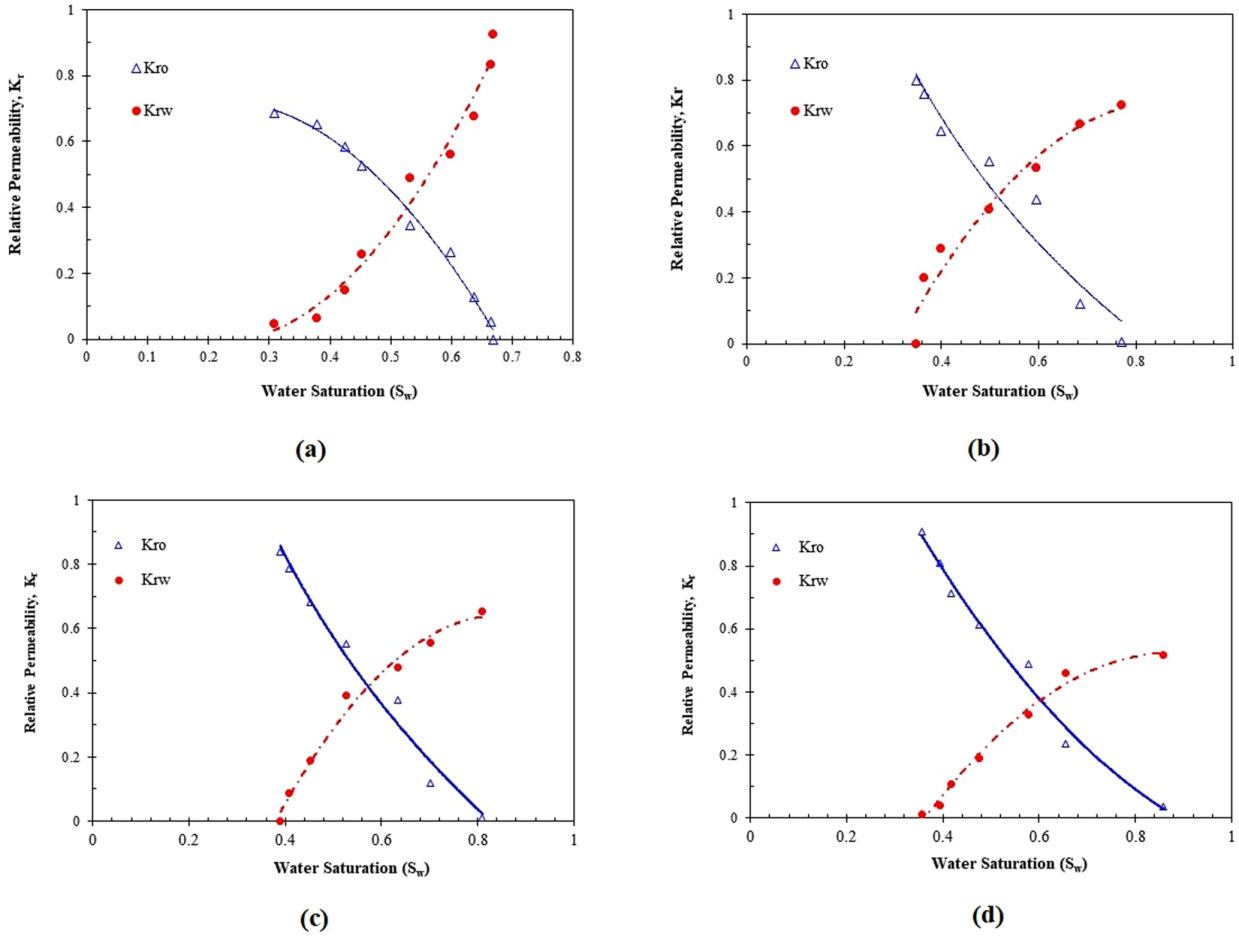
Water and oil relative permeability curves for (**a**) brine flooding, (**b**) 4 wt% surfactant, (**c**) 6 wt% surfactant, (**d**) 8 wt% surfactant.

**Figure 16:**
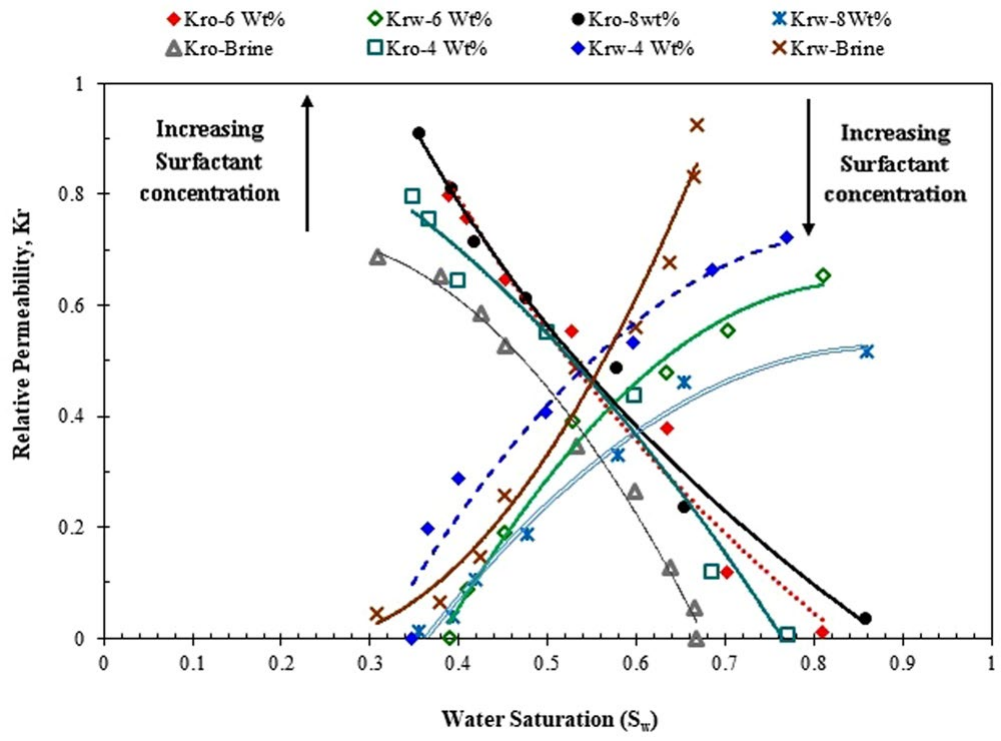
Comparison between water and oil relative permeability curves for coreflooding tests with different ZSC concentrations.

According to Toth *et al*. (2006) method^33^, the oil and water relative permeabilities (*K*_*ro*_ & *K*_*rw*_) are determined based on the data generated throughout the core displacement experiments, as exhibited in Fig. 15. As shown in Fig. 15a, the cross-point of the water and oil relative permeability (*K*_*ro*_) curves is observed at S_w_ = 0.5, while the oil relative permeability (*K*_*rw*_) at irreducible water saturation is 0.6867 and the water relative permeability at the residual oil saturation is 0.92445. Relative permeability (*K*_*r*_) curves for the core flooding of 4 wt% surfactant are depicted in Fig. 15b. As clear from Fig. 15b, the cross-point of the relative permeability curves is at S_w_ = 0.58, whereas the oil relative permeability at the irreducible water saturation and water relative permeability at the residual oil saturation are 0.7975 and 0.7235, respectively. The cross-points of the relative permeabilities and also end point relative permeability of other concentrations of ZSC are found in Fig. 15c,d. Figure 16 combines all panels of Fig. 15 for a better comparison in terms of end-point saturation relative permeability and the cross -points. As demonstrated in Fig. 16, the residual oil saturation lowers as the surfactant content of aqueous phase increases. Furthermore, increasing the surfactant concentration of the solution results in an increase in the magnitude of the oil relative permeability and a reduction in water relative permeability.

### Practical Implication

The efficiency of water/surfactant flooding strongly depends on several parameters such as fluids properties, rock characteristics, and their interactions. Effects of salinity on the displacement process and adsorption have been recognized as an important aspect in production processes by chemical and petroleum researchers while injecting water/surfactant into petroleum reservoirs. Effective and economical oil recovery throughout waterflooding needs an in-depth understanding of the mechanism of adsorption phenomenon, a variation of adsorption loss, the behavior of relative permeability functions and oil saturation distribution at various values of salinity and surfactant concentration. This study assists petroleum researchers and engineers understand the above key features of water/oil/brine systems in terms of adsorption density and recovery factor through a systematic and comprehensible manner. Due to the high importance of salinity in surfactant consumption and mobility ratio during waterflooding, this phase of research is instructive to choose an apposite surfactant with a proper concentration for EOR utilization as well as reservoir stimulation in the oil reservoirs. It also helps to determine the optimum salinity with respect to economic and technical prospects attributed to waterflooding processes.

## Conclusions

Through an experimental investigation, adsorption of a new natural surfactant derived from the leaves of ZSC onto carbonate rock samples was studied at various salinity values. An attempt was also made to find out the adsorption mechanisms through examining different isotherm models. Beside static adsorption tests, the core flooding experiments were carried out to investigate the effect of ZSC on oil and water relative permeabilities. Based on the study outcomes, the following conclusions can be drawn:

1. The presence of salt in water/surfactant flooding process increases the positive charge of the carbonates, resulting in an increase in the attraction forces and consequently the higher quantity of adsorption loss.
2. Given the experimental data, the highest adsorption density is observed for the sample containing MgCl_2_. The conclusion is valid for the whole range of the salt concentrations tested in the experiments.
3. For all types of salts used in this study, increasing the salt concentration causes an increase in the amount of adsorption fate on the carbonate surface.
4. The real adsorption data exhibits an acceptable agreement with the Freundlich equilibrium model as high *R*^2^ (>0.98) and low error percentage are obtained.
5. Provided important information from the adsorption kinetics runs, the pseudo-second-order model is followed by the experimental data when ZSC is adsorbed on the surface of carbonates.
6. Based on the dynamic tests, adding ZSC surfactant to brine considerably enhances oil recovery due to an increase in the oil relative permeability.

## Electronic supplementary material

Supporting Information
