# Supplementary material for: Spotlight on the New Natural Surfactant Flooding in Carbonate Rock Samples in Low Salinity Condition
Source: Sci Rep. 2018 Jul 20;8:10985. doi: 10.1038/s41598-018-29321-w (PMC6054617; doi:10.1038/s41598-018-29321-w)
Supplement: Supplementary file 1 — Supporting Information [file 41598_2018_29321_MOESM1_ESM.docx]

**Supporting Information**

Spotlight on the New Natural Surfactant Flooding in Carbonate Rock Samples in Low Salinity Condition

Mohammad Ali Ahmadi^1*^, Seyed Reza Shadizadeh^2^

^1)^ Department of Chemical and Petroleum Engineering, University of Calgary, Calgary, Alberta, Canada

^2)^ Department of Petroleum Engineering, Petroleum University of Technology, Abadan, Iran.

^*)^ Address to Corresponding Author: Department of Chemical and Petroleum Engineering, University of Calgary, Calgary, Alberta, Canada. Email: [mohammadali.ahmadi@ucalgary.ca](mailto:mohammadali.ahmadi@ucalgary.ca), TEL: 001-587-2889700

**Solution Preparation (Static Tests).** As the first stage, the surfactant solution was prepared within the concentration range of 1000 mg/L- 80000mg/L. These solutions were then diluted to obtain standard solutions containing 1000, 5000, 10000, 15000, 20000, 40000, 50000, 60000, 70000 and 80000 mg/L of the natural surfactant. Solutions of the surfactant with different salinity between 0 mg/L to 10000 mg/L were supplied by dissolving 0.0-1.0 g of each type of salt in 100 mL of the surfactant solutions.

**Adsorption Isotherm Models**

This section summarizes the results and discussion for the static isotherms which are not appropriate to model adsorption behaviour of ZSC employed for carbonate samples.

**Langmuir Isotherm:** Figure S1 demonstrates the fitting of the adsorption data to the Langmuir adsorption isotherm when different salinities are tried. Table S1 is also obtained using Figure S1 and statistical analysis to present the values of the important parameters for the Langmuir model and the correlation coefficient (*R^2^*) under different conditions (e.g., salt concentration).

As explained in the Section Theory of the paper (e.g. Adsorption Models), the reverse of equilibrium adsorption density (1/*q_e_*) versus the reverse of equilibrium surfactant concentration (1/*C_e_*) is plotted as depicted in Figure S1 based on Langmuir model. The slop of the fitted straight line represents (1/*q_o_*🞨*K_ad_*) and intercept of the line with vertical axis stands for (1/*q_o_*Another crucial index for the Langmuir isotherm model is the term “*R_L_*” which is a non-dimensional constant, called as separation factor or equilibrium parameter. This factor is defined as follows ^23,24^:

| $R_{L}=\frac{1}{1+K_{ad} C_{e}}$ | (S1) |
| --- | --- |

There are various ranges for R_L_ as the following:

• When 0 < *R_L_* < 1, the adsorption process is favorable.

• If *R_L_* > 1, the adsorption process is unfavorable.

• *R_L_* = 1 represents linear trend of adsorption behavior.

• *R_L_* = 0 demonstrates that the adsorption process is irreversible.

The value of R_L_ obtained for this work varies from 0.0985 to 0.944. This confirms favorably of the adsorption process of surfactant onto carbonate rock faces.

**Temkin Isotherm:** Assuming the adsorption data follow the Temkin isotherm model, Figure S2 demonstrates the adsorption behavior. In addition, Table S2 also lists the Temkin parameters along with the magnitudes of *R^2^* for the linear equations. To determine the Temkin isotherm parameters, equilibrium adsorption density (q_e_) versus Ln (C_e_) is plotted as depicted in Figure S2. Since very low values for *R^2^* are obtained based on the experimental data, the Temkin model is not a proper isotherm to predict adsorption density onto the carbonate reservoir rock while using ZSC as the natural and bio-degradable surfactant.

**Linear model:** Presenting the adsorption data on the basis of the linear isotherm model (e.g., the amount of adsorption loss, *q*, versus equilibrium concentration, *C_e_*), the slop of the straight line stands for the K_H_. Also, the intercept of the fitted line represents the constant of the linear model. The fitting of the experimental adsorption data to the linear isotherm model results in Figure S3.

Based on the results demonstrated in Figure S3, the parameters of the linear isotherm model for all aqueous systems are summarized in Table S3. As clear from the table, the correlation coefficient (*R^2^*) is about 0.94 which is higher than that for the Temkin isotherm, but lower than *R^2^* for the Freundlich and Langmuir models.

| (a)  (b)  (c) |
| --- |
| **Figure S1:** Langmuir adsorption model of natural surfactant adsorption onto carbonates, a) at different KCl concentrations, b) at different NaCl concentrations, c) at different MgCl_2_ concentrations |

**Table S1:** Parameters of the Langmuir adsorption model employed in this study at different salinity

| **MgCl_2_ (PPM)** | **Correlation** | ***R^2^*** | ***q_o_*** | ***K_ad_*** |
| --- | --- | --- | --- | --- |
| 0 | 1/ q_e_ = 0.049/Ce + 0.0291 | 0.9897 | 34.364 | 0.593 |
| 1000 | 1/ q_e_ = 0.0355/Ce + 0.0336 | 0.9715 | 29.761 | 0.946 |
| 5000 | 1/ q_e_ = 0.0274/Ce + 0.0337 | 0.9658 | 29.673 | 1.229 |
| 10000 | 1/ q_e_ = 0.0263/Ce + 0.0301 | 0.9734 | 33.222 | 1.144 |
| **KCl (PPM)** |  | | | |
| 1000 | 1/ q_e_ = 0.0371/Ce + 0.0345 | 0.9732 | 28.985 | 0.929 |
| 5000 | 1/ q_e_ = 0.0342/Ce + 0.0342 | 0.9689 | 29.239 | 1.000 |
| 10000 | 1/ q_e_ = 0.0335/Ce + 0.0318 | 0.9728 | 31.446 | 0.949 |
| **NaCl (PPM)** |  | | | |
| 1000 | 1/ q_e_ = 0.0345/Ce + 0.0355 | 0.9664 | 28.169 | 1.028 |
| 5000 | 1/ q_e_ = 0.0319/Ce + 0.0334 | 0.972 | 29.94 | 1.047 |
| 10000 | 1/ q_e_ = 0.0276/Ce + 0.032 | 0.9696 | 31.25 | 1.159 |

| (a)  (b)  (c) |
| --- |
| **Figure S2:** Temkin isotherm model of natural surfactant adsorption onto carbonates, a) at different KCl concentrations, b) at different NaCl concentrations, c) at different MgCl_2_ concentrations |

**Table S2:** Parameters of Temkin adsorption model employed in this study

| **MgCl_2_ (PPM)** | **Correlation** | **R^2^** | ***K_t_*** | ***B*** |
| --- | --- | --- | --- | --- |
| 0 | q_e_ = 11.5ln(Ce) + 20.811 | 0.8202 | 6.1083 | 11.5 |
| 1000 | q_e_ = 12.362ln(Ce) + 22.637 | 0.8169 | 6.2412 | 12.362 |
| 5000 | q_e_ = 12.524ln(Ce) + 23.574 | 0.8177 | 6.5686 | 12.524 |
| 10000 | q_e_ = 13.581ln(Ce) + 25.556 | 0.8147 | 6.5649 | 13.581 |
| **KCl (PPM)** |  | | | |
| 1000 | q_e_ = 11.898ln(Ce) + 21.743 | 0.817 | 6.2180 | 11.898 |
| 5000 | q_e_ = 12.326ln(Ce) + 22.602 | 0.816 | 6.2569 | 12.326 |
| 10000 | q_e_ = 12.955ln(Ce) + 23.854 | 0.8174 | 6.3047 | 12.955 |
| **NaCl (PPM)** |  | | | |
| 1000 | q_e_ = 12.074ln(Ce) + 22.09 | 0.8155 | 6.2310 | 12.074 |
| 5000 | q_e_ = 12.312ln(Ce) + 22.881 | 0.8176 | 6.4136 | 12.312 |
| 10000 | q_e_ = 13.093ln(Ce) + 24.511 | 0.8138 | 6.5017 | 13.093 |

| (a)(b)  (c) |
| --- |
| **Figure S3:** Linear isotherm model of natural surfactant adsorption onto carbonates, a) at different KCl concentrations, b) at different NaCl concentrations, c) at different MgCl_2_ concentrations |

**Table S3:** Parameters of Linear adsorption model employed in this study

| **MgCl_2_ (PPM)** | **Correlation** | **R^2^** | ***K_H_*** | ***C*** |
| --- | --- | --- | --- | --- |
| 0 | q_e_ = 7.3423Ce + 4.4132 | 0.9433 | 7.3423 | 4.4132 |
| 1000 | q_e_ = 7.9979Ce + 4.8219 | 0.9436 | 7.0079 | 4.8219 |
| 5000 | q_e_ = 8.1168 Ce + 5.5294 | 0.9402 | 8.1168 | 5.5294 |
| 10000 | q_e_ = 8.922 Ce + 5.751 | 0.945 | 8.922 | 5.751 |
| **KCl (PPM)** |  | | | |
| 1000 | q_e_ = 7.6549 Ce + 4.6634 | 0.9437 | 7.6549 | 4.6634 |
| 5000 | q_e_ = 7.9731 Ce + 4.8318 | 0.944 | 7.9731 | 4.8318 |
| 10000 | q_e_ = 8.4365 Ce + 5.107 | 0.943 | 8.4365 | 5.107 |
| **NaCl (PPM)** |  | | | |
| 1000 | q_e_ = 7.7932 Ce + 4.7041 | 0.9444 | 7.7932 | 4.7041 |
| 5000 | q_e_ = 7.9484 Ce + 5.2 | 0.9385 | 7.9484 | 5.2 |
| 10000 | q_e_ = 8.5415 C_e_ + 5.543 | 0.9385 | 8.5415 | 5.543 |

**General structure of Saponin**

Figure S4 shows the general structure of the saponin, representative of ZSC that acts as a surfactant.

**Figure S4:** schematic of the saponin's structure^30,31^
